# Genome-Wide SNP Calling from Genotyping by Sequencing (GBS) Data: A Comparison of Seven Pipelines and Two Sequencing Technologies

**DOI:** 10.1371/journal.pone.0161333

**Published:** 2016-08-22

**Authors:** Davoud Torkamaneh, Jérôme Laroche, François Belzile

**Affiliations:** 1 Département de Phytologie, Université Laval, Quebec City, QC, Canada; 2 Institut de Biologie Intégrative et des Systèmes (IBIS), Université Laval, Quebec City, QC, Canada; Universidad Miguel Hernández de Elche, SPAIN

## Abstract

Next-generation sequencing (NGS) has revolutionized plant and animal research in many ways including new methods of high throughput genotyping. Genotyping-by-sequencing (GBS) has been demonstrated to be a robust and cost-effective genotyping method capable of producing thousands to millions of SNPs across a wide range of species. Undoubtedly, the greatest barrier to its broader use is the challenge of data analysis. Herein we describe a comprehensive comparison of seven GBS bioinformatics pipelines developed to process raw GBS sequence data into SNP genotypes. We compared five pipelines requiring a reference genome (TASSEL-GBS v1& v2, Stacks, IGST, and Fast-GBS) and two *de novo* pipelines that do not require a reference genome (UNEAK and Stacks). Using Illumina sequence data from a set of 24 re-sequenced soybean lines, we performed SNP calling with these pipelines and compared the GBS SNP calls with the re-sequencing data to assess their accuracy. The number of SNPs called without a reference genome was lower (13k to 24k) than with a reference genome (25k to 54k SNPs) while accuracy was high (92.3 to 98.7%) for all but one pipeline (TASSEL-GBSv1, 76.1%). Among pipelines offering a high accuracy (>95%), Fast-GBS called the greatest number of polymorphisms (close to 35,000 SNPs + Indels) and yielded the highest accuracy (98.7%). Using Ion Torrent sequence data for the same 24 lines, we compared the performance of Fast-GBS with that of TASSEL-GBSv2. It again called more polymorphisms (25.8K vs 22.9K) and these proved more accurate (95.2 vs 91.1%). Typically, SNP catalogues called from the same sequencing data using different pipelines resulted in highly overlapping SNP catalogues (79–92% overlap). In contrast, overlap between SNP catalogues obtained using the same pipeline but different sequencing technologies was less extensive (~50–70%).

## Introduction

Next-generation sequencing (NGS) has facilitated greatly the development of methods to genotype very large numbers of molecular markers such as single nucleotide polymorphisms (SNPs). NGS offers several approaches that are capable of simultaneously performing genome-wide SNP discovery and genotyping in a single step, even in species for which little or no genetic information is available [1]. This revolution in genetic marker discovery enables the study of important questions in molecular breeding, population genetics, ecological genetics and evolution. The most highly used methods of genotyping relying on NGS use restriction enzymes to capture a reduced representation of a genome [2–9]. New approaches such as restriction site-associated DNA sequencing (RAD-seq) and genotyping-by-sequencing (GBS) have been developed as rapid and robust approaches for reduced-representation sequencing of multiplexed samples that combines genome-wide molecular marker discovery and genotyping [1]. This family of reduced representation genotyping approaches generically called genotyping-by-sequencing (GBS) [1]. The flexibility and low cost of GBS makes this an excellent tool for many applications and research questions in genetics and breeding. Such modern advances allow for the genotyping of thousands of SNPs, and, in doing so, the probability of identifying SNPs correlated with traits of interest increases [10]. Even with advancement of NGS to produce millions of sequence reads per run, data analysis for these new approaches can be complex owing to using restriction enzymes, sample multiplexing, different fragment length and variable read depth [1]. It is crystal clear that advanced analysis pipelines have become a necessity to filter, sort and align this sequence data. A pipeline for GBS must include steps to filter out poor-quality reads, classify reads by pool or individuals based on sequence barcodes, either identify loci and alleles *de novo* or align reads to an index reference genome to discover polymorphisms, and often score genotypes for each individual included in the study. Generally, pipelines for handling GBS data are categorized in two groups; *de novo*-based and reference-based. When a reference genome is available, the reads from reduced-representation sequencing can be mapped to the reference genome and SNPs can be called as for whole-genome resequencing projects [11–12]. Up to now, several reference-based GBS analysis pipelines have been developed. The most widely used reference-based GBS analysis pipelines are: TASSEL-GBS (v1 and v2), Stacks, IGST, and Fast-GBS (the most recent pipeline, Torkamaneh et al. (unpublished)) [9, 13–15]. In the absence of a reference genome, pairs of nearly identical reads (presumed to represent alternative alleles of a locus) need to be identified. The most highly used pipelines for such a *de novo*-based approach are UNEAK and Stacks [15, 16].

Finally, different NGS sequencing platforms are currently available and offer different advantages. For example, whereas the Illumina technology offers very high throughput and read quality, this usually comes at the expense of speed as close to two weeks are required to complete a run. In contrast, the Ion Torrent technology [17] offers great speed (4 hours) at the expense of lower throughput and read quality. Depending on the constraints, one or the other technology may prove more suitable. Ideally, one would like SNP calling pipelines to perform equally well with both types of read data.

In this study, we comprehensively compared existing GBS analysis pipelines on the basis of the number of SNPs called, the accuracy of the resulting genotypes as well as the speed and ease of use of these pipelines. We also compared the results obtained using Illumina and Ion Torrent reads. Finally, we examined the amount of overlap in the SNP loci that were called using different pipelines.

## Materials and Methods

### Samples and sequencing platform

Soybean (*Glycine max L*.) is a diploid species with 20 pairs of chromosomes and it has a medium-sized genome (1.1 Gb). Because it is an autogamous species, soybean lines/cultivars breed true and are highly homozygous. A set of 23 Canadian soybean lines and one plant introduction (PI) was subjected to GBS analysis. These same lines were resequenced as previously described by Torkamaneh and Belzile [18]. Using the same DNA, two GBS libraries were constructed following *Ape*KI digestion: one for Illumina sequencing (as per Elshire et al. [6]) and the other for Ion Torrent sequencing (as per Mascher et al. [19]). Single-end sequencing was performed either on an Illumina HiSeq 2000 at the McGill University-Génome Québec Innovation Center in Montreal, Canada, or on an Ion Proton machine at the Institut de Biologie Intégrative et des Systèmes (IBIS) of Université Laval, Quebec, Canada. A total of 42 million 100-bp reads were generated on the Illumina platform and 38 million 50- to 135-bp reads were obtained on the Ion Torrent platform. All data (GBS and WGS) are available in the NCBI Sequence Read Archive (SRA) under accessions SRP059747 (Illumina sequences) and SRP073237 (Ion Torrent sequences).

### GBS analysis pipelines

We used two *de novo* variant callers and five reference-based pipelines (Williams82 reference genome; [20]) to call SNPs. We ran all pipelines in the same conditions of depth of coverage (minDP≥2), maximum mismatch for alignment (n = 3), Maximum Missing Data (MaxMD = 80%), and Minimum Minor Allele Frequency (MinMAF≥0.05). Below, we briefly describe the processes for each pipeline. For computation, we used a Linux system with 10 CPU and 25G of memory. In addition to the descriptions provided below, a summary of the different components of each pipeline is provided in S1 Table and we provide all command lines used in this work as supporting information (S1 Text).

#### Fast-GBS

The Fast-GBS analysis pipeline has been developed by integrating public packages with internally developed tools. The core functions include: (1) demultiplexing and cleaning of raw sequence reads; (2) read quality assessment and mapping; (3) filtering of mapped reads and estimation of library complexity; (4) re-alignment and local haplotype construction; (5) fit population frequencies and individual haplotypes; (5) raw variant calling; (6) variant and individual-level filtering; (7) identification of highly consistent variants. Since researchers may not always have immediate access to cluster resources, this pipeline allows either parallel processing of a large number of samples in a cluster or serial processing of multiple samples on a single machine.

#### IGST (IBIS Genotyping-by-Sequencing Tool)

A pipeline implemented in Perl programming language was developed for the processing of Illumina sequence read data. The steps involved in the pipeline were executed in separate shell scripts. This pipeline uses different publicly available software tools (FASTX toolkit, BWA, SAMtools, VCFtools) as well as some in-house tools [11, 21, 22]. The raw SNPs obtained were further filtered using VCFtools based on read depth, missing data in genotypes and minor allele frequency. Heterozygous correction is performed by an in-house Python script.

#### TASSEL-GBS (version 1 and 2)

TASSEL-GBS pipelines are implemented in Java programming language. Currently, two versions are available: TASSEL-GBS v1 (TASSEL 3.0) [13] and TASSEL-GBS v2 (TASSEL 5.0) [14]. Both pipelines function in a similar manner and require that all reads be trimmed to an identical length (64 bp in v1, up to 92 bp in v2) and identical reads are collapsed into tags. These tags are then aligned against the reference genome and SNPs are called from aligned tags. The main changes implemented in TASSEL-GBS v2 are: 1) the possibility to use longer tags to improve the accuracy of alignment to the reference genome and 2) an enhanced SNP discovery and production step.

#### UNEAK (Universal Network Enabled Analysis Kit)

The general design of UNEAK is as follows: 1) reads are trimmed to 64 bp; 2) identical 64-bp reads are collapsed into tags; 3) pairwise alignment identifies tag pairs having a single base pair mismatch. These single base pair mismatches are candidate SNPs. A “network filter” is employed to discard repeats, paralogs and sequencing errors, resulting in a collection of reciprocal tag pairs, or SNPs.

#### Stacks (reference-based and *de novo*)

The raw input data to Stacks are sequenced DNA fragments from any restriction enzyme–based GBS protocol. Stacks can handle raw sequencing data to identify loci *de novo* or via alignment against a reference genome [10]. Regardless of whether the data are assembled *de novo*, or aligned against a reference genome, many subsequent steps in Stacks are shared. The pipeline can be described as follows: (1) Raw sequence reads are demultiplexed and cleaned (process_radtags). (2) Data from each individual are grouped into loci, and polymorphic nucleotide sites are identified (ustacks or pstacks for unaligned or aligned data, respectively). (3) Loci are grouped together across individuals and a catalogue is written (cstacks). (4) Loci from each individual are matched against the catalogue to determine the allelic state at each locus in each individual (sstacks). (5) Allelic states are either converted into a set of mappable genotypes (for a genetic map) using genotypes or subjected to population genetic statistics via populations, with the results being written in one or several output files.

### Genotype accuracy

For the estimation of the accuracy of genotype calls, we used an in-house script to compare the genotypes called using GBS with the genotypes called at the same loci following WGS. The sequencing and calling of SNPs in this collection of 24 soybean lines was previously described in Torkamaneh and Belzile [18]. Briefly, soybean lines were sequenced to a mean depth of coverage of 9x and a genome coverage of 96% was achieved. Illumina paired-end reads were aligned onto the soybean reference genome (Williams82) using BWA and the genotypes at polymorphic loci were called using SAMtools. Variants with two or more alternative alleles were removed. A total of 3.6M SNPs were thus called among these lines. As a complementary means to measure genotype quality, we estimated the proportion of missing data and heterozygous calls produced with each analysis pipeline. For *de novo* pipelines we aligned the tags supporting SNPs against reference genome to find the physical position and then we compared them with WGS dataset.

## Results

### Variant calling with different pipelines using Illumina read data

To assess the performance of different GBS analysis pipelines, we analyzed publicly available GBS data (100-bp Illumina reads) from a set of 24 previously studied soybean lines. We compared five reference-based analysis pipelines: TASSEL-GBS v1 and v2, Stacks, IGST, and Fast-GBS. We also compared two widely used *de novo* variant callers: UNEAK and Stacks. We used the same number of reads for all analyses (42M reads) and attempted to select parameters that would be as similar as possible for all the pipelines (see M&M for details). As shown in Table 1, large differences in the number of SNPs called were seen with both *de novo* and reference-based pipelines. Among the former, Stacks called the fewest SNPs, ~2 fold fewer than UNEAK (13,303 vs 24,743). The number of SNPs called by UNEAK was not too far below the mean number of SNPs called by reference-based pipelines (32,423). Among reference-based pipelines, the number of SNPs called varied between 18,941 (Stacks) and 54,412 (TASSEL-GBS v1), a 2.8-fold difference. The other three reference-based pipelines were much closer to the mean, calling between roughly 25k and 35k SNPs. In addition to calling SNPs, IGST and Fast-GBS were also able to call indels. In both cases, these contributed an extra 12–13% to the tally of variants.

**Table 1:** Number of SNPs and indels detected among 24 soybean lines using seven different bioinformatics pipelines on Illumina reads. The time and amount of memory needed to run each pipeline are also provided.

		Variants			
Approach	Pipeline	SNPs	Indels	Time* (h:m)	Memory (Gb)
***de novo***	Stacks	13,303	ND	3:07	7
	UNEAK	24,743	ND	1:11	20
**Reference- based**	TASSEL-GBSv1	54,412	ND	1:45	15
	Stacks	18,941	ND	3:30	14
	IGST	25,650	3,170	12:59	240
	TASSEL-GBSv2	28,158	ND	4:16	18
	Fast-GBS	34,953	3,921	1:47	27

* Using a Linux system with 10 CPU and 25G of memory

Fast-GBS and TASSEL-GBS v1 proved to be the fastest running among the reference-based pipelines (~1h45), whereas IGST proved the slowest, requiring almost 13h to complete the analysis. Among *de novo* pipelines, UNEAK was almost three times faster than Stacks (1h11 vs 3h07) and proved the fastest of all pipelines. In terms of memory required, here also, very large differences were observed. Among *de novo* pipelines, UNEAK required almost three times as much disk space compared to Stacks (20 Gb vs 7 Gb). Among the reference-based pipelines, the differences were even greater as IGST required 17.1-fold more memory (240 Gb) than Stacks (14 Gb).

### Accuracy and efficacy of GBS bioinformatics pipelines

To examine the quality of the SNP data obtained using reference-based pipelines, we first measured the amount of missing data and then estimated genotype accuracy by comparing the GBS-derived genotypes with the true genotypes uncovered through whole-genome resequencing of the same lines. Assessments of the accuracy of GBS-called SNPs were performed on all SNPs for all pipelines at the same levels of tolerance for missing data (≤80%) and minor allele frequency (≥0.05). As can be seen in Table 2, among reference-based pipelines, the proportion of missing data varied from as little as 28% (TASSEL GBS v1) to as much as 57.3% (Stacks). Among the *de novo* pipelines, the proportion of missing data was less variable, ranging from 39.4% (Stacks) to 41.3% (UNEAK).

**Table 2:** Accuracy of GBS SNP data derived from Illumina platform using different bioinformatics pipeline.

Approach	*de novo*		Reference-based				
Parameter/Pipeline	Stacks	UNEAK	TASSEL-GBS v1	Stacks	IGST	TASSEL-GBS v2	Fast-GBS
Number of SNPs	13,303	24,743	54,412	18,941	25,650	28,158	34,953
Number of genotypes	319,272	593,832	1,305,888	454,584	615,600	675,792	838,872
Missing data (%)	41.3	39.4	28	57.3	44	35.6	46
Heterozygotes (%)	3.7	5.3	11.5	4.4	5.9	5.7	3.4
Loci with >50% heterozygotes*	0	0	1125	65	324	551	184
Accuracy (%)	93.6	93.9	76.1	93.2	98.4	92.3	98.7

*These were eliminated from the final catalogue used to estimate accuracy

When we compared the genotypes obtained using each pipeline with the genotypes derived from resequencing, we found that 98.7% of SNP genotypes called using the Fast-GBS pipeline matched the true genotypes. Similar levels of accuracy were found for SNPs called with IGST (98.4%). With a single exception, all reference-based pipelines achieved levels of accuracy >92%. TASSEL-GBS v1 proved the least accurate of these pipelines, as only 76.1% of the genotypes it called were identical to the resequencing data. Among *de novo* pipelines, the accuracy of genotype calls was only slightly lower (93.7%, on average) than that obtained with the reference-based pipelines other than TASSEL-GBS v1 (95.6%, on average).

Among plants, recent or ancient polyploidization events can generate paralogs that can be mistaken to represent alleles of a single locus based on short sequence reads. We therefore examined both the overall number of heterozygous genotype calls and the number of loci containing a large proportion (>50%) of heterozygous calls. As can be seen in Table 2, *de novo* pipelines called a similar proportion of heterozygous genotypes (~3.7 and 5.3% for Stacks and UNEAK, respectively), and did not retain any loci with a large proportion of heterozygotes. Among reference-based pipelines, Fast-GBS and TASSEL-GBS v1 called the fewest and the most heterozygous genotypes (3.4 and 11.5%, respectively). Additionally, TASSEL-GBS v1 called the largest number of loci with a large proportion of heterozygous genotypes (1125), while Stacks only called 65 loci with more than 50% heterozygotes.

### Overlap between SNP catalogues

We then determined the degree of overlap between the SNP catalogues obtained using the different pipelines and their accuracy. We selected Fast-GBS as the basis for comparison because of its ability to very accurately call a large number of SNPs. As demonstrated in Table 3, among reference-based pipelines, the most overlap was observed between Fast-GBS and Stacks (>96%), and 92% of SNPs called with IGST were also found in the Fast-GBS dataset. In contrast, TASSEL-GBS v1 showed the lowest overlap (36.7%) with Fast-GBS. The *de novo* pipelines showed similar levels of overlap with Fast-GBS (Stacks = 89.1% and UNEAK = 87.5%). In an additional analysis (not shown in Table 3), we measured the overlap between the two *de novo* pipelines; around 67% of SNPs called by Stacks were also found in the UNEAK dataset. These two *de novo* pipelines therefore seem to identify fairly distinct subsets of the more extensive SNP catalog obtained using Fast-GBS.

**Table 3:** Degree of overlap among SNP loci called using Fast-GBS and six other bioinformatics pipelines

		SNPs			
Approach	Pipeline	Total	Common (in %)	Other pipeline only	Fast-GBS only
***de novo***	Stacks	13,303	89.1	1,450	23,100
	UNEAK	24,743	87.5	3,172	13,382
**Reference-based**	TASSEL-GBS v1	54,412	36.7	34,420	14,961
	Stacks	18,941	96.2	1,709	16,721
	IGST	25,650	92.4	1,950	11,253
	TASSEL-GBS v2	28,158	88.3	3,295	10,090

**Fig 1:**
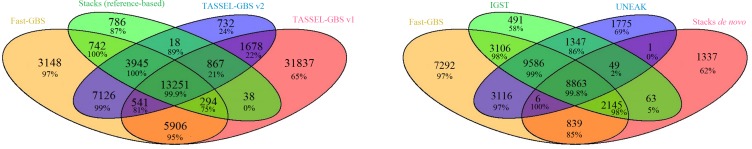
Venn diagram representing the degree of overlap among SNP loci called using seven bioinformatics pipelines. The percentages indicate the estimated accuracy for all groups of SNPs (unique or shared).

To gain a deeper understanding of the genotypic accuracy among different subsets of shared or unique SNPs, we prepared two separate Venn diagrams, each comprising only four pipelines (for clarity), with Fast-GBS included in both panels (Fig 1). What stands out in this figure is that SNPs called by more than one pipeline were typically highly accurate (weighted mean accuracy = 94.8%). In contrast, with the sole exception of Fast-GBS, SNPs called by a single pipeline were typically much less accurate (weighted mean accuracy = 66.3%). Most strikingly, we note that TASSEL-GBS v1 called a very large number of unique SNPs (over 30,000) that show a low accuracy (65%). Unique SNPs called by other pipelines also typically showed low accuracy but were far fewer in number and thus had less impact overall.

### Reasons for poor performance of some pipelines

**Fig 2:**
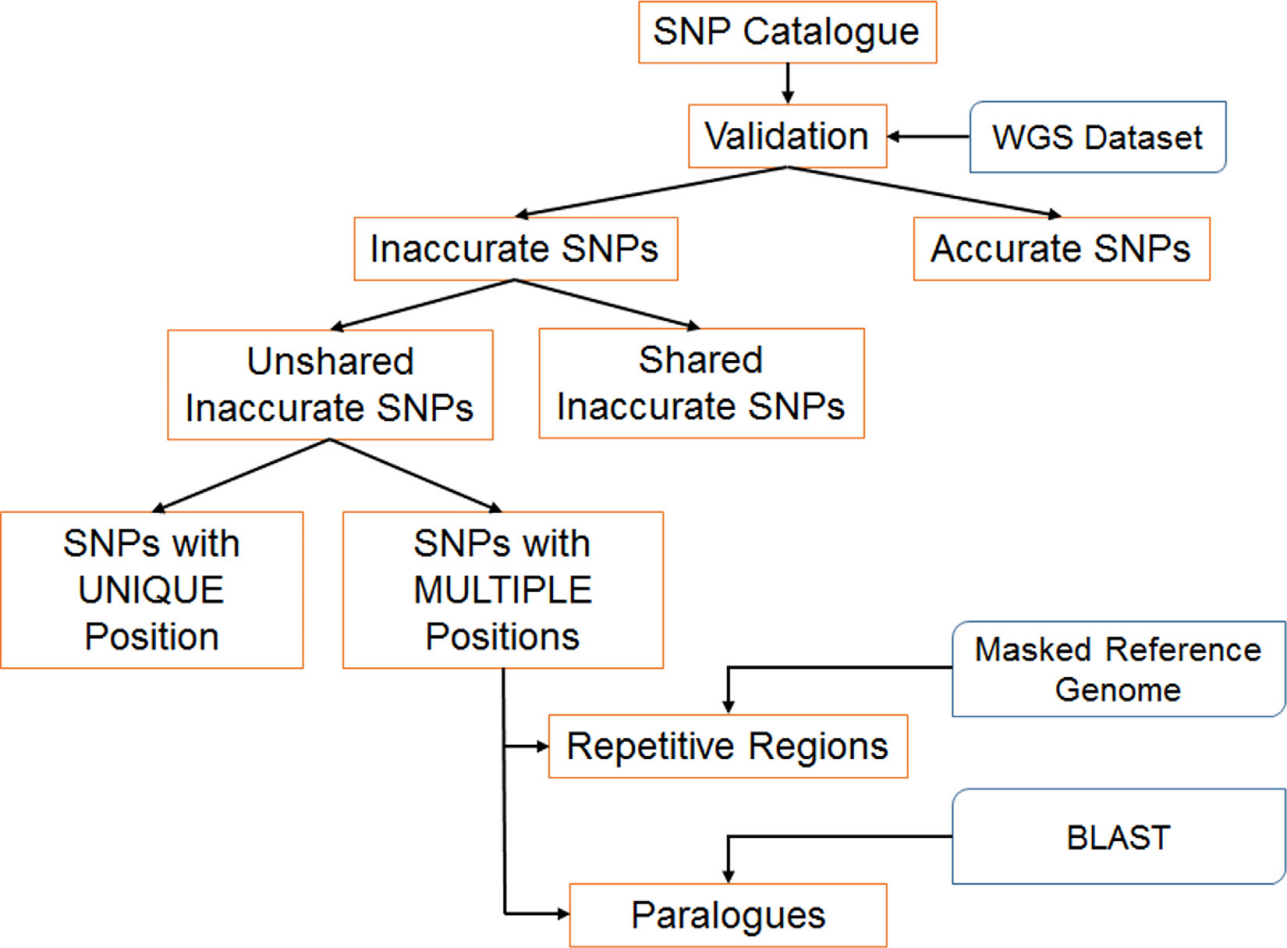
Systematic approach used to investigate the possible causes of unique inaccurate SNP calls.

Given the observed variation in the number of called SNPs and their accuracy, we chose to investigate the causes of erroneous calls. To conduct this investigation, we followed a systematic approach illustrated in Fig 2. We divided the catalogue of SNPs in two categories, accurate and inaccurate, based on the comparison of the GBS-derived calls and the calls resulting from WGS. Inaccurate SNPs were then classified as being either unique to a single pipeline or shared between at least two pipelines. To investigate unique “weaknesses” of pipelines, we focused our attention on unique inaccurate SNPs. The first step in this investigation was to classify these inaccurate SNPs as being supported by reads mapping to a unique position in the genome or by reads mapping to multiple positions. In the first case, genotyping errors were attributed to a fault by the variant caller (e.g. due to sequencing or PCR amplification errors). In the second case, we reasoned that the mapping of reads to more than one location in the genome could result from these reads originating from either paralogues or repetitive regions. To resolve this, we mapped the reads against the masked reference genome (Phytozome V9: Gmax-189-hardmasked.fa) to estimate the proportion of inaccurate SNPs originating from repetitive regions. SNPs that were no longer present in the catalogue derived from mapping to the masked reference genome were taken to be due to repetitive sequences. The remaining reads that successfully mapped to multiple sites in the masked reference genome were analyzed via a BLAST search to detect paralogy. A read was deemed to derive from a paralogue when we encountered at least 2 hits with 100% coverage and minimum of 96% identity. On average, reads originating from paralogous loci (as defined above) had 2.4 hits in the genome.

The results of this analysis are shown in Table 4. As most pipelines provided a largely accurate (>92%) set of SNPs, only a few hundred unique inaccurate SNPs were called by each pipeline with the sole exception of TASSEL-GBS v1 (9,828 unique inaccurate SNPs). A minority (11.5 to 29.7%) of the unique inaccurate SNPs were supported by reads mapping to a single position in the genome and deemed to result from an error in variant calling. The majority (70.3 to 88.5%) of inaccurate SNPs were supported by reads mapping to more than one region in the genome. Among these, the vast majority were due to reads mapping to paralogous regions (74 to 93%). We therefore conclude that most genotyping errors in soybean could be attributed to the presence of paralogs and that TASSEL-GBS v1 proved to be, by far, the pipeline most subject to making erroneous calls because of this.

**Table 4:** Number and characteristics of unique inaccurate SNPs called by different pipelines.

*Approach*	*de novo*		*Reference-based*				
*Pipeline*	Stacks	UNEAK	TASSEL GBS v1	Stacks	IGST	TASSEL GBS v2	Fast-GBS
***Unique inaccurate SNPs***	495	533	9,828	103	207	558	272
	(3.7% of 13,303)	(2.2% of 24,743)	(18.1% of 54,412)	(0.5% of 18,941)	(0.8% of 25,650)	(2.0% of 28,158)	(0.8% of 34,953)
***Inaccurate SNPs with unique position (% of unique inaccurate SNPs)***	146	72	1,126	20	46	132	35
	(29.7)	(13.5)	(11.5)	(19.4)	(22.2)	(23.7)	(12.9)
***Inaccurate SNPs with multiple positions (% of unique inaccurate SNPs)***	349	461	8,702	83	161	426	237
	(70.3)	(86.5)	(88.5)	(80.6)	(77.8)	(76.3)	(87.1)
***Repetitive region (% of inaccurate SNPs with multiple positions)***	45	120	1,828	9	15	60	17
	(13)	(26)	(21)	(11)	(9)	(14)	(7)
***Paralogues (% of inaccurate SNPs with multiple positions)***	304	341	6,875	74	146	366	220
	(87)	(74)	(79)	(89)	(91)	(86)	(93)

Another result that begged investigation was the relatively low number of SNPs called by Stacks, as both *de novo* and reference-based versions of Stacks had called the fewest SNPs. We investigated the efficacy of the demultiplexing step as this had already been described as problematic. In our analyses, we found that 19.7% of Illumina reads failed to be assigned to a specific barcode file, a number that is much higher than that seen with the other pipelines. To measure the impact of such a decrease in the number of reads available to call SNPs, we used an alternative demultiplexing tool (Sabre), instead of the one provided in Stacks. The proportion of missing reads decreased to ~2% and the number of SNPs called using this more extensive set of reads increased by 12 and 24% (21,456 and 17,342) for Stacks reference-based and Stacks *de novo*, respectively. We conclude that the poor performance of the Stacks demultiplexing tool is an important contributor to the decreased number of SNPs called by Stacks.

### GBS using different sequencing platforms

To compare SNP calling using different sequencing technologies, we performed GBS on the same 24 soybean samples on an Ion Torrent platform. In contrast to Illumina reads that are all exactly the same length (100 bp), Ion Torrent reads varied in length from 50 to 135 bp. In this analysis, we used only two reference-based pipelines that had performed best in the tests described above (Fast-GBS and TASSEL-GBS v2) using 38 million Ion Torrent reads. As seen in Table 5, the number of SNPs called with each pipeline at the same levels of tolerance for missing data (≤80%) and minor allele frequency (≥0.05) was highly similar (~23K in both cases). As above, Fast-GBS called a greater number of variants as it called a total of over 2,000 indels in addition to the SNPs. In terms of computing time, Fast-GBS was more than two-fold faster than TASSEL-GBS v2 (1h31 vs 3h29), while it used 15% more disk space (20 Gb vs 17 Gb).

**Table 5:** Number of SNPs and indels detected among 24 soybean lines using Ion Torrent reads and two different bioinformatics pipelines

		Variants			
Approach	Pipeline	SNP	Indels	Time* (h:m)	Memory (Gb)
**Reference- based**	TASSEL-GBSv2	22,921	ND	3:29	17
	Fast-GBS	23,792	2,054	1:31	20

* Using a Linux system with 10 CPU and 25G of memory

In a second analysis, we measured the amount of missing data and estimated the accuracy of genotypes both by comparing GBS-called genotypes to the ones obtained through resequencing and by assessing the amount of heterozygosity in these lines that are presumed homozygous. As can be seen in Table 6, the proportion of missing data was relatively similar for the two pipelines (37% vs 33%). In this analysis TASSEL-GBS v2 called more heterozygous genotypes than Fast-GBS (6.6% vs 4.5%). Also TASSEL-GBS v2 called many more loci with a large proportion (>50%) of heterozygous genotypes than Fast-GBS (4,831 vs 861). In this analysis, Fast-GBS again achieved the highest accuracy in calling genotypes (95.2%), compared to 91.1% using TASSEL-GBS v2.

**Table 6:** Accuracy of SNP data derived using Ion Torrent reads and two different bioinformatics pipelines

Stat type/Pipeline	TASSEL-GBSv2	Fast-GBS
**Number of SNPs**	22,921	23,792
**Missing data (%)**	37	33
Loci with >50% heterozygotes*	4,831	861
**Residual heterozygotes (%)**	6.6	4.5
**Accuracy (%)**	91.1	95.2

*These were eliminated from the final catalogue used to estimate accuracy

**Fig 3:**
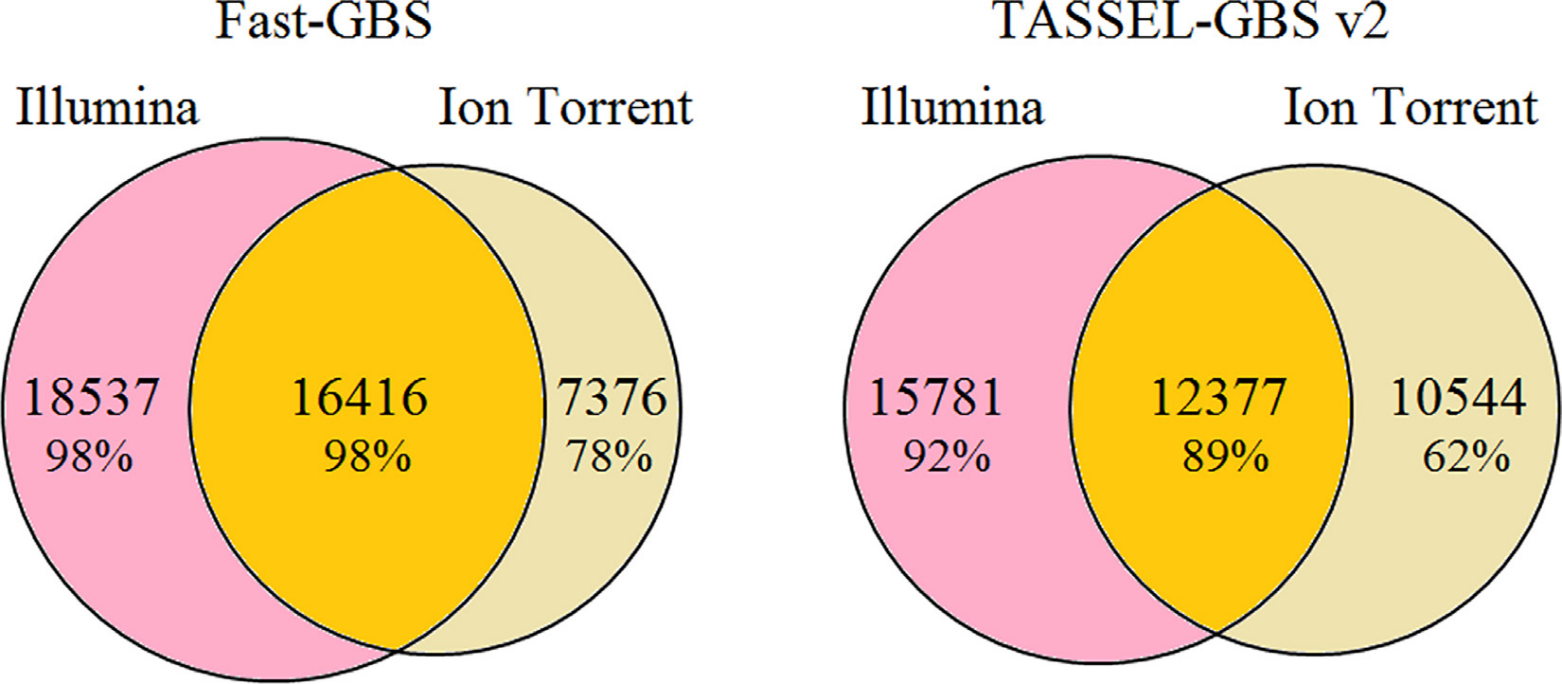
Venn diagram for overlap of the SNPs called using two different bioinformatics pipelines (a) Overlap of SNPs called with Fast-GBS using Illumina and Ion Torrent reads. (b) Overlap of SNPs called with TASSEL-GBS v2 using Illumina and Ion Torrent reads. The percentages indicate the estimated accuracy for all groups of SNPs (unique or shared).

Finally, we compared the overlap among SNP catalogues obtained using the two sequencing platforms (Illumina vs Ion Torrent). As illustrated in Fig 3, when using Fast-GBS, we found that 69% (16,416 of 23,792 SNPs) of the SNPs derived from Ion Torrent reads were also present in the catalogue of SNPs obtained using Illumina reads. Conversely, of all the SNPs called using Illumina reads (34,953 SNPs), 47% were in common with the Ion Torrent catalogue. Using TASSEL-GBS v2, a slightly lower proportion (54%) (12,377 of 22,921 SNPs) of SNPs called from Ion Torrent reads were also obtained using Illumina reads. Conversely, a similar proportion (44%) of SNPs called using Illumina reads were in common with those called using the Ion Torrent reads. We found that using Ion Torrent reads leads to a greater number of inaccurate SNPs compared to Illumina reads. Using Illumina reads, only 23.7% and 12.9% of inaccurate SNPs called by TASSEL-GBS v2 and Fast-GBS had a unique position, while using Ion Torrent reads this proportion increased to 76% and 87% for TASSEL-GBS v2 and Fast-GBS, respectively. On the other hand, the number of inaccurate SNPs due to paralogy and repetitive regions were similar for both technologies. Based on these results, we conclude that the observed increase in the number of inaccurate SNPs with a unique position (not due to any sort of repetitive sequence) is due to the higher frequency of sequencing errors in Ion Torrent reads.

In conclusion, the amount of overlap across sequencing platforms was similar using both pipelines but much lower than the overlap seen across pipelines using the same sequencing platform.

## Discussion

The flexibility and low cost of genotyping methods relying on NGS make these excellent tools for many applications and research questions in genetics, breeding, and biodiversity [3, 6, 23–25]. Currently, GBS appears to be favored in the agricultural sciences (plant and animal breeding) whereas RAD-Seq seems to be the more prevalent approach in the field of ecology [1]. Whatever library preparation approach is chosen to achieve complexity reduction prior to sequencing, bioinformatics must be used to extract useful information on SNP loci and genotypes from a vast amount of short sequence reads [1, 26]. It is at this stage that the choice of an analytical method will have the greatest impact on the amount and quality of the resulting genotypic information. Unfortunately, to date, few studies have systematically compared SNP-calling pipelines for GBS and compared their efficiency, accuracy and degree of overlap.

The first question that arises concerns the use of *de novo* vs reference-based methods. In the absence of a reference genome, there is little choice but to use one of the two currently widespread tools, UNEAK and Stacks. Although they use different algorithms to do so, these two pipelines are conceptually similar in that they seek to first establish catalogues of identical reads and then to search for highly related reads that are potentially alleles at the same locus. Under the conditions used in this work, UNEAK greatly outperformed Stacks in that it generated 82% more SNPs (~25k vs ~13k). From a qualitative perspective, both *de novo* pipelines performed similarly well in terms of missing data (~40%) and genotypic accuracy (~94%). This is comparable to the results reported by Lu et al. (2013) in maize where it was estimated that 92% of genotype calls were accurate and that this proportion could be increased to 96.2% by filtering for SNPs with a MAF > 0.3 in a segregating biparental population [16]. Both *de novo* pipelines can be run quite quickly and are relatively conservative in their SNP calls resulting in a dataset of high quality. Thus, for the vast majority of species for which no reference genome is available currently or in the foreseeable future, the *de novo* SNP calling tools perform extremely well in terms of accuracy, but UNEAK will yield almost two-fold more SNPs.

The picture painted of the performance of *de novo* pipelines in this comparison may be too rosy, however. Indeed, for the sake of uniformity, we used the same filtering options (MinMAF≥0.05, MaxMD = 80%, and minDP≥2) for both *de novo* and reference-based pipelines. But this high tolerance towards missing data may not be realistic in the case of *de novo* pipelines. We have shown previously that missing data imputation is very efficient and accurate on a dense set of SNPs obtained using a reference-based pipeline [18]. In the case of *de novo* pipelines, in the absence of positional information on the different SNPs and the haplotype structure, imputation is much more challenging. For this reason, most users of *de novo* pipelines will set a lower ceiling for the maximal amount of missing data, typically between 20% and 50% at most [16, 19, 27]. With the GBS sequence data used in this work, tolerating up to 20% of missing data substantially decreases the number of SNPs that can be called using both *de novo* pipelines (~5k SNPs; data not shown). Under these more realistic conditions (in view of the necessary imputation of missing data), we find that reference-based pipelines yielded about 5- to 7-fold more high-quality SNP markers (~5k vs 25k to 35k markers).

Given the increasing availability of reference genomes in economically important crops and animals, we then need to ask which of the available reference-based pipelines produces the best catalogue of SNPs both in terms of abundance of markers and their accuracy. Among the five reference-based pipelines, Fast-GBS can be run quickly, resulted in the highest genotyping accuracy for a very large number of SNP loci (close to 35,000) in addition to almost 4,000 indels. Based on these considerations, it seems to be the pipeline of choice, at least in the case of soybean and likely also for other species with similar genomic and reproductive characteristics.

Of the pipelines tested, TASSEL-GBSv1 stood out from the rest of the group in terms of the number of SNP loci called (50–100% more than the others), but this came at the cost of accuracy as it was the only pipeline whose genotypic calls were accurate in less than 90% of cases (76.1%). As it is not easy to distinguish true from false genotypes, we would argue that TASSEL-GBSv1 is insufficiently accurate to be used on its own. In previous work, the large resulting catalogue of SNPs was often “filtered” by discarding markers that did not behave as expected in a segregating population [6]. This presumably helped to discard “false” markers that resulted from confounding alleles (at a single locus) and reads derived from paralogous loci. We hypothesized that the main reason for this decreased accuracy is the fact that TASSEL-GBSv1 clips all reads to a uniform length of 64 bases, thus producing short tags that are at increased risk of mapping to multiple or erroneous locations. Pipelines using longer reads did not exhibit this problem and typically had at least 10-fold fewer reads mapping to multiple locations. For example, despite sharing much in common with TASSEL-GBS v1, when TASSEL-GBS v2 was run under conditions that allow for longer tags (92 bases in our case), the reliability of the genotypes increased considerably.

The reference-based version of Stacks is the other pipeline that stood out in that it called much fewer SNPs than the others. In investigating the different steps needed to go from sequences to SNPs, we found that Stacks lost ~20% of reads at the demultiplexing step, i.e. some barcoded reads were not attributed to a sample and were simply discarded from the ensuing steps. This obviously resulted in a concomitant decrease in the number of SNPs called (~19k vs ~25k). This poor performance of the Stacks demultiplexing step has been previously reported by Herten et al [28].

In our view, the genome-wide measurement of the accuracy of GBS datasets derived from different bioinformatics pipelines represents an important and key contribution of this work. It was assessed by comparing directly to whole genome resequencing data. In many previous studies, estimates of genotypic accuracy were often achieved by indirect measurement [16] or performed on a very small subset of SNP loci [9]. Typically, levels of genotype accuracy ranging between 92 and 98% have been reported with slight differences being observed between species and types of population [9, 16, 19]. The advantage of using resequencing data in this fashion is that we can directly assess the accuracy of GBS data yielded by different pipelines.

Another important consideration is whether the SNP catalogues produced using different pipelines and different sequencing technologies are concordant. When using a single sequencing technology (Illumina), we found that ~80% or more of SNPs called by most pipelines were also present in the SNP catalogue derived from Fast-GBS. Thus, these pipelines largely agree on the loci that are polymorphic within a given set of germplasm. The only exception was TASSEL-GBS v1, as, only a quarter of the SNPs present in the resulting catalogue was also present in the set derived using Fast-GBS. This is likely due to the shorter sequences used (only 64 bp) and a large number of “false” SNPs as this pipeline proved the least accurate of all. When using the same pipeline to analyze data derived from two sequencing technologies (Illumina and Ion Torrent), we typically found that the overlap between SNP catalogues varied between roughly 50 and 70%. Thus, the choice of sequencing technology used resulted in a greater variability in the catalogue of SNPs produced than did the choice of pipeline used on a single set of reads. At first glance, this would seem to contradict the conclusions drawn by Mascher et al. (2013) who found that the SNP catalogues produced using two pipelines (TASSEL-GBS v1 and SAMtools) differed more than the catalogues obtained using different sequencing technologies (Illumina and Ion Torrent) [19]. In our view, this is more a reflection of the limitations of TASSEL-GBS v1 (due to its short tags). When we consider a broader array of reference-based pipelines, these generally provide a very good overlap in SNP loci uncovered.

The conclusions drawn from this work are likely to extend to other organisms sharing similar genomic features (medium-sized genome, diploid). It can be anticipated that species having experienced recent whole genome duplication events will represent a greater challenge as the risk of confounding alleles at the same locus and paralogs will likely increase in such cases. In species where such events occurred in the more distant past, there will have been more opportunity for paralogs to diverge, thus facilitating the correct mapping of reads.

As such, it is impossible to devise a single pipeline that will be equally suited to every situation. This is where it becomes important for users to be able to change various parameters in the SNP calling process. Unfortunately, not all pipelines are equally “transparent” in this regard and offer the same opportunity to be altered. At one end of the spectrum, UNEAK and TASSEL-GBS offer very good performance, but rely on some purpose-built tools or algorithms that a user cannot easily alter (e.g. for demultiplexing and variant calling). Also, the intermediate data files are not always easily accessible and this makes it more difficult to investigate specific problems. At the other end of the spectrum, IGST and Fast-GBS string together a set of existing tools for which the user can alter parameters/options at will, and the intermediate files are easily accessible. In this spectrum, in our view, Stacks offers an intermediate level of transparency.

Finally, although whole-genome sequencing of entire populations is rapidly approaching, we believe that the methods described here are likely to remain invaluable for years to come in population genomics, breeding, mapping studies and reference genome sequence assembly, particularly for non-model organisms.

## Supporting Information

S1 Table Summary of five reference based GBS pipelines. (DOCX)

S1 Text Command lines for seven pipelines used in this study. (DOCX)
