# Supplementary material for: Genome-Wide SNP Calling from Genotyping by Sequencing (GBS) Data: A Comparison of Seven Pipelines and Two Sequencing Technologies
Source: PLoS One. 2016 Aug 22;11(8):e0161333. doi: 10.1371/journal.pone.0161333 (PMC4993469; doi:10.1371/journal.pone.0161333)
Supplement: S1 Table — (DOCX) [file pone.0161333.s001.docx]

| **Step/Pipeline** | **TASSEL-GBS v1** | **Stacks** | **IGST** | **TASSEL-GBS v2** | **Fast-GBS** |
| --- | --- | --- | --- | --- | --- |
| **Demultiplexer** | FasqToTagCount | Stacks | BarcodeSplitter | GBSSeqToTagDB | Sabre |
| **Trimmer** | FasqToTagCount | Cutadapt | FASTX-Toolkit | GBSSeqToTagDB | Cutadapt |
| **Read/Tag** | Tag | Read | Read | Tag | Read |
| **Minimum/Maximum Read/Tag length (bp)** | 32-64 | >50 | >50 | 20-92 | >50 |
| **Aligner** | BWA-aln | BWA-mem | BWA-aln | BWA-aln | BWA-mem |
| **Post-processing of mapped reads** | No | No | No | No | Yes |
| **Variant caller** | GBSTagToSNP | pstacks | SAMtools | DiscoverySNPCaller v2 | Platypus |
| **Single/Multi-sample variant calling** | Single | Multi | Single | Single | Multi |

**S1 Table.** Summary of five reference-based GBS pipelines. For each pipeline, we indicate the tools and conditions used to carry out the major steps that are needed to produce a catalogue of SNPs starting from reads in a FASTQ file.
