# Supplementary material for: Genome-Wide SNP Calling from Genotyping by Sequencing (GBS) Data: A Comparison of Seven Pipelines and Two Sequencing Technologies
Source: PLoS One. 2016 Aug 22;11(8):e0161333. doi: 10.1371/journal.pone.0161333 (PMC4993469; doi:10.1371/journal.pone.0161333)
Supplement: S1 Text — (DOCX) [file pone.0161333.s002.docx]

**S1 Text. Command lines for seven pipelines used in this study.**

For the two pipelines requiring a parameter file (IGST and Fast-GBS), the “default” parameter file that is provided for each pipeline was used. For pipelines that do not require a parameter file (Stacks, UNEAK, TASSEL-GBS v1 and v2), the default parameters were used except where indicated otherwise in the M&M. The numbers within square brackets throughout the document indicate the source references and they are found at the end of this document.

**Fast-GBS**

export PRG=/FastGBS/FastGBS.sh **[1]**

export PRG=/make_directories.sh

export PARAM=/fastGBS/fastGBS_parameter.txt

$PRG $PARAM

------------------------------------------------------------------

**IGSTsamt**

export PRG=/igst/IGSTsamt.pl **[2]**

export PARAM=/igst/IGSTsamt-parameter.txt

$PRG $PARAM

------------------------------------------------------------------

**TASSEL-GBS-v1 (TASSEL 3)**

------------------------------------------------------------------

Step 1: FastqToTagCountPlugin

run_pipeline.pl **[3]** -fork1 -FastqToTagCountPlugin -e ApeKI -i $FASTQ -k $KEY -o $TAG -endPlugin -runfork1

------------------------------------------------------------------

Step 2: MergeMultipleTagCountPlugin

run_pipeline.pl -fork1 -MergeMultipleTagCountPlugin -i $TAG -o $MERGE/masterTags -c 2 -t -endPlugin -runfork1

run_pipeline.pl -fork1 -MergeMultipleTagCountPlugin -i $TAG -o $MERGE/masterTags.cnt -c 2 -endPlugin -runfork1

------------------------------------------------------------------

Step 3: BWA_Alignment

bwa aln -n 3 -k 1 -t 10 $REF/Gmax_109_chr.fa $MERGE/masterTags.fq > $MERGE/masterTags.sai

bwa samse -n 1 $REF/Gmax_109_chr.fa $MERGE/masterTags.sai $MERGE/masterTags.fq > $MERGE/masterTags.sam

------------------------------------------------------------------

Step 4: SAMConverterPlugin

run_pipeline.pl -fork1 -SAMConverterPlugin -i $MERGE/masterTags.sam -o $TOPM/masterTags.topm.bin -endPlugin -runfork1

------------------------------------------------------------------

Step 5: FastqToTBTPlugin:

run_pipeline.pl -fork1 -FastqToTBTPlugin -i $FASTQ -k $KEY -e ApeKI -o $TBT -t $MERGE/masterTags.cnt -y -endPlugin -runfork1

------------------------------------------------------------------

Step 6: MergeTagsByTaxaFilesPlugin

run_pipeline.pl -fork1 -MergeTagsByTaxaFilesPlugin -i $TBT -o $MTBT/glycine24.tbt.bin -endPlugin -runfork1

------------------------------------------------------------------

Step 7: TagsToSNPByAlignmentMTPlugin

run_pipeline.pl -fork1 -TagsToSNPByAlignmentMTPlugin -i $MTBT/glycine24.tbt.bin -m $TOPM/masterTags.topm.bin -o $HAPU -s 1 -e 20 -endPlugin -runfork1

------------------------------------------------------------------

Step 8: MergeDuplicateSNPsPlugin

run_pipeline.pl -fork1 -MergeDuplicateSNPsPlugin -hmp $HAPU/mergedTBT.c+.hmp.txt -o $HAPM/mergedTBT.mergedSNPs.c+.hmp.txt -sC 1 -eC 20 -endPlugin -runfork1

------------------------------------------------------------------

Step 9: GBSHapMapFiltersPlugin

run_pipeline.pl -fork1 -GBSHapMapFiltersPlugin -hmp $HAPM/mergedTBT.mergedSNPs.c+.hmp.txt -o $HAPF/mergedTBT.mergedSNPs.filt.c+.hmp.txt -sC 1 -eC 20 -endPlugin -runfork1

-----------------------------------------------------------------

**TASSEL-GBS-v2 (TASSEL 5)**

------------------------------------------------------------------

Step 1: GBSSeqToTagDBPlugin

run_pipeline.pl **[4]** -fork1 -GBSSeqToTagDBPlugin -e $ENZ -i $FASTQ -db $DB -k $KEY -mxTagL $MX -mnTagL $MN -mnQS $QS -endPlugin -runfork1

------------------------------------------------------------------

Step 2: TagExportToFastqPlugin

run_pipeline.pl -fork1 -TagExportToFastqPlugin -db $DB -o $TAG -c 2 -endPlugin -runfork1

------------------------------------------------------------------

Step 3:BWA_Alignment

bwa aln -n 3 -k 1 -t 10 $REF $TAG > $SAM.sai

bwa samse -n 1 $REF $SAM.sai $TAG > $SAM.sam

------------------------------------------------------------------

Step 4:SAMToGBSdbPlugin

run_pipeline.pl -fork1 -SAMToGBSdbPlugin -i $SAM.sam -db $DB -aProp 0.0 -aLen 0 -endPlugin -runfork1

------------------------------------------------------------------

Step 5:DiscoverySNPCallerPluginV2

run_pipeline.pl -fork1 -DiscoverySNPCallerPluginV2 -db $DB -sC 1 -eC 20 -ref $REF -mnLCov 0.1 -mnMAF 0.01 -endPlugin -runfork1

------------------------------------------------------------------

Step 6:SNPQualityProfilerPlugin

run_pipeline.pl -fork1 -SNPQualityProfilerPlugin -db $DB -statFile $STAT -endPlugin -runfork1

------------------------------------------------------------------

Step 7:ProductionSNPCallerPluginV2

run_pipeline.pl -fork1 -ProductionSNPCallerPluginV2 -db $DB -e $ENZ -i $FASTQ -k $KEY -mxTagL $MX -mnQS $QS -o $HAP -endPlugin -runfork1

------------------------------------------------------------------

**Stacks-reference-based [5]**

------------------------------------------------------------------

Step 1: cutadapt

./00-scripts/01_cutadapt.sh **[6]**

------------------------------------------------------------------

Step 2: process_radtags

./00-scripts/02_process_radtags.sh 92 apeKI

------------------------------------------------------------------

Step 3: rename_samples

./00-scripts/03_rename_samples_complex.sh

------------------------------------------------------------------

Step 5: pstacks

./00-scripts/stacks_1a_pstacks.sh

------------------------------------------------------------------

Step 6: cstacks

./00-scripts/stacks_2_cstacks.sh

------------------------------------------------------------------

Step 7: sstacks

./00-scripts/stacks_3_sstacks.sh

------------------------------------------------------------------

Step 8: populations

./00-scripts/stacks_4_populations.sh

------------------------------------------------------------------

**Stacks-de-novo [5]**

------------------------------------------------------------------

Step 1: cutadapt

./00-scripts/01_cutadapt.sh **[6]**

cutadapt -a adaptor sequence input.fastq > output.fastq

------------------------------------------------------------------

Step 2: process_radtags

./00-scripts/02_process_radtags.sh 92 apeKI

------------------------------------------------------------------

Step 3: rename_samples

./00-scripts/03_rename_samples_complex.sh

------------------------------------------------------------------

Step 5: pstacks

./00-scripts/stacks_1a_pstacks.sh

------------------------------------------------------------------

Step 6: cstacks

./00-scripts/stacks_2_cstacks.sh

------------------------------------------------------------------

Step 7: sstacks

./00-scripts/stacks_3_sstacks.sh

------------------------------------------------------------------

Step 8: populations

./00-scripts/stacks_4_populations.sh

------------------------------------------------------------------

**UNEAK**

UCreatWorkingDirPlugin

run_pipeline.pl **[7]** -fork1 -UCreatWorkingDirPlugin -w $DIR -endPlugin -runfork1

------------------------------------------------------------------

Step 1: UFastqToTagCountPlugin

run_pipeline.pl -fork1 -UFastqToTagCountPlugin -w $DIR -e ApeKI -endPlugin -runfork1

------------------------------------------------------------------

Step 2: UMergeTaxaTagCountPlugin

run_pipeline.pl -Xms10G -Xmx20G -fork1 -UMergeTaxaTagCountPlugin -w $DIR -c 5 -endPlugin -runfork1

------------------------------------------------------------------

Step 3: UTagCountToTagPairPlugin

run_pipeline.pl -Xms10G -Xmx20G -fork1 -UTagCountToTagPairPlugin -w $DIR -e 0.03 -endPlugin -runfork1

------------------------------------------------------------------

Step 4: UTagPairToTBTPlugin

run_pipeline.pl -Xms10G -Xmx20G -fork1 -UTagPairToTBTPlugin -w $DIR -endPlugin -runfork1

------------------------------------------------------------------

Step 5: UTBTToMapInfoPlugin

run_pipeline.pl -Xms10G -Xmx20G -fork1 -UTBTToMapInfoPlugin -w $DIR -endPlugin -runfork1

------------------------------------------------------------------

Step 6: UMapInfoToHapMapPlugin

run_pipeline.pl -Xms10G -Xmx20G -fork1 -UMapInfoToHapMapPlugin -w $DIR -endPlugin -runfork1

------------------------------------------------------------------

**References:**

1. <https://bitbucket.org/jerlar73/fastgbs>. Access contact: [Jerome.Laroche@ibis.ulaval.ca](mailto:Jerome.Laroche@ibis.ulaval.ca)
2. <https://openi.nlm.nih.gov/detailedresult.php?img=PMC3553054_pone.0054603.g001&req=4>. Access contact: [Jerome.Laroche@ibis.ulaval.ca](mailto:Jerome.Laroche@ibis.ulaval.ca)
3. <https://sourceforge.net/p/tassel/tassel3-standalone/ci/master/tree/run_pipeline.pl>
4. <https://sourceforge.net/p/tassel/tassel5-standalone/ci/master/tree/run_pipeline.pl>
5. <http://catchenlab.life.illinois.edu/stacks/>
6. <https://cutadapt.readthedocs.io/en/stable/guide.html#basic-usage>
7. <https://sourceforge.net/p/tassel/tassel3-standalone/ci/master/tree/run_pipeline.pl>
